# Modulation of Quorum Sensing and Biofilms in Less Investigated Gram-Negative ESKAPE Pathogens

**DOI:** 10.3389/fmicb.2021.676510

**Published:** 2021-07-29

**Authors:** Veronica Lazar, Alina Maria Holban, Carmen Curutiu, Mariana Carmen Chifiriuc

**Affiliations:** ^1^Department of Microbiology and Immunology, Faculty of Biology, University of Bucharest, Bucharest, Romania; ^2^The Research Institute of the University of Bucharest, Bucharest, Romania

**Keywords:** ESKAPE, microbial biofilms, intercellular communication, quorum sensing inhibitors, quorum quenching, personalized therapy

## Abstract

Pathogenic bacteria have the ability to sense their versatile environment and adapt by behavioral changes both to the external reservoirs and the infected host, which, in response to microbial colonization, mobilizes equally sophisticated anti-infectious strategies. One of the most important adaptive processes is the ability of pathogenic bacteria to turn from the free, floating, or planktonic state to the adherent one and to develop biofilms on alive and inert substrata; this social lifestyle, based on very complex communication networks, namely, the quorum sensing (QS) and response system, confers them an increased phenotypic or behavioral resistance to different stress factors, including host defense mechanisms and antibiotics. As a consequence, biofilm infections can be difficult to diagnose and treat, requiring complex multidrug therapeutic regimens, which often fail to resolve the infection. One of the most promising avenues for discovering novel and efficient antibiofilm strategies is targeting individual cells and their QS mechanisms. A huge amount of data related to the inhibition of QS and biofilm formation in pathogenic bacteria have been obtained using the well-established gram-positive *Staphylococcus aureus* and gram-negative *Pseudomonas aeruginosa* models. The purpose of this paper was to revise the progress on the development of antibiofilm and anti-QS strategies in the less investigated gram-negative ESKAPE pathogens *Klebsiella pneumoniae*, *Acinetobacter baumannii*, and *Enterobacter* sp. and identify promising leads for the therapeutic management of these clinically significant and highly resistant opportunistic pathogens.

## Introduction

Many international organizations have declared antibiotic resistance (AR) to be a global public health concern, requiring concerted action plans to tackle this problem and to decrease its huge social, medical, and economic burden (Michael et al., 2014; Spellberg et al., 2016).

The emergence of AR strains is favored by microbiostatic substances, which only inhibit microbial multiplication, but also by the improper administration of microbicidal drugs with respect to dose interval and active concentration (Vrancianu et al., 2020a,c). Some of the multiple negative consequences of AR are the unprecedented increase in infectious disease frequency, illness duration, morbidity and mortality rates, as well as associated costs and failure of performing medical procedures requiring effective antibiotic prophylaxis and treatment, such as organ transplantation, cancer therapy, major surgery, management of preterm babies, and use of implanted medical devices (Laxminarayan et al., 2013; Weist and Diaz Högberg, 2014).

A pivotal but still underestimated contribution to the dimension of AR problem is brought by microbial biofilms developed on cellular/tissue substrates or medical devices (Lazãr and Chifiriuc, 2010; Pircalabioru and Chifiriuc, 2020). These microbial communities or biofilms represent a form of existence with a particular architecture and behavior, different from that of single, free-floating, or planktonic cells living in citadels challenging to conquer (Lazãr and Chifiriuc, 2010; Lazar, 2011), which is also more advantageous for bacteria, mainly due to the intercellular communication and sense of their density inside biofilms and exhibiting high phenotypic resistance (or tolerance) to high doses of antimicrobial agents. According to the National Institutes of Health (Bethesda, MD, United States), biofilm-associated infections (BAIs) are involved in the etiology of 70 – 80% of human infections (Bjarnsholt et al., 2005; Lazar, 2011). In 2000, the CDC (Center for Disease Control, United States) stated that BAIs are one of the seven major healthcare safety challenges for the medical community, which has to find solutions for reducing catheter-associated infections as well as hospitalization and mortality from respiratory tract infections that occur in long-term care patients (Thomas et al., 2006; Rice, 2010).

One of the most important traits ensuring the success of pathogenic strains in establishing persistent infections is their intricate and very complex communication systems, involving group-specific or interchangeable signaling molecules, used to coordinate growth, virulence, as well as bacteriocins and antibiotics production (González and Keshavan, 2006; Jayaraman and Wood, 2008; Lazar, 2011; Stoica et al., 2016). The most studied intra-, interspecies, and even interkingdom communication system is the quorum sensing (QS) and response mechanism, mediated by small chemical signals, called autoinducers (AIs). If at the beginning of the 1990s the QS mechanism was known only for *Vibrio fischeri* and *Vibrio harveyi* (Fuqua et al., 1994) and is considered an “interesting but esoteric” mechanism of gene regulation, only a few years later, using a lux-based reporter gene screening model, it was described in most gram-negative bacteria (Passador and Iglewsi, 1995). QS metabolites are produced by both prokaryotic and eukaryotic cells for one-way, two-way, or multiway communication (Jayaraman and Wood, 2008; Kendall and Sperandio, 2014), in response to bacterial population changes and environmental cues (e.g., starvation, hypoxia, low iron availability) (Lee and Zhang, 2015; Ha et al., 2018). It has been shown that pathogenic QS molecules could alter the host microbiota and also interfere with host cell signaling pathways (Curutiu et al., 2013; Vogt et al., 2015). Moreover, bacterial pathogens can recognize and utilize various mammalian molecules, such as hormones (epinephrine and norepinephrine), interleukins, and signaling peptides (Freestone et al., 2000; Curutiu et al., 2013). The most common types of AIs used by gram-negative bacteria in intraspecies communication are the *N*-acyl homoserine lactones (AHLs) (Watson et al., 2002), while gram-positive bacteria use autoinducing peptides (AIPs) (Jayaraman and Wood, 2008).

Among many important advantages offered by QS to bacterial pathogens, there is the ability to colonize and/or invade the host, as well as to develop biofilms on natural tissues (skin, mucosa, endothelial epithelia, and teeth) or medical devices (central venous catheters, peritoneal, urinary catheters, dental materials, cardiac valves, intrauterine contraceptive devices, contact lenses, and other implants) (Lazãr and Chifiriuc, 2010) and, thus, to persist in the host.

Quorum sensing signaling is involved in key points of the biofilm development (initiation, matrix formation, maturation, and detachment) and modulates collective phenotypes responsible for biofilm structure, such as surface motility and the production of exopolysaccharides (EPSs) and other adhesins (Hooshdar et al., 2020). Currently investigated approaches for BAI control include (i) bacteriophages (Neguţ et al., 2014), (ii) mechanical debridement of biofilms by ultrasound and surgical procedures, (iii) biophysical approaches to facilitate drug penetration and/or delivery inside biofilms (infrared and light pulsing, direct-current electrical stimulation, ultrasound and alternating electric fields, etc.) (Kim, 2016), (iv) drug delivery systems (Kasimanickam et al., 2013), (v) local delivery of antibiotics (including the revived ones, such as colistin) in high concentrations for a long period of time (e.g., catheter locks, intratracheal locks, etc.) (Chauhan et al., 2012), (vi) antipathogenic (antivirulence) molecules, (vii) new types of vaccines using cells with the adhesive phenotype (Lazar, 2011), (viii) matrix dispersing/degrading/destabilizing agents [enzymes, anti-EPS antibodies, nucleic acid binding proteins, and ethylenediaminetetraacetic acid (EDTA)] (Kolderman et al., 2015), (ix) targeting non-growing dormant and persister biofilm cells (Conlon et al., 2013), and (x) development of modified biomedical devices, resistant to microbial adhesion and colonization (Abdelghany et al., 2019).

The disruption of bacterial QS by QS inhibitors (QSIs) represent a promising approach for fighting BAIs (Davies et al., 1998; Chandra Kalia, 2013; Fong et al., 2018). As most of the described QS signaling systems include two-component systems (TCS), namely, the AI (QS molecule) and the receptor, also known as response regulator (RR), which impacts on the transcription of target genes (Papenfort and Bassler, 2016), the QS modulation strategies follow one of two directions: (i) interference with signal generation and (ii) signal reception (Zhao et al., 2020). Both directions cluster various approaches and are summarized in Table 1. However, in some situations, the AI (i.e., AI-3) can bind to a sensor kinase (SK), instead of an RR (Kim et al., 2020).

**TABLE 1:** Mechanisms of quorum sensing modulation in Gram negative bacteria.

Main approach	Mechanism	Type/Target	Result	References
Signal generation	Inhibition of the AI synthesis	LuxI inhibitor	Inhibition of AHL synthesis	Chan et al., 2015
		SAM (S adenozyl methionine) inhibitor	Inhibition of AI-2 synthesis	Taylor et al., 2009
Signal reception	Degradation of the AI	Lactonases	Open the ring of AHLs – inactive AI	See-Too et al., 2018
		Acylases	Cut the lateral acyl chain of AHLs – inactive AI	Utari et al., 2017
	Receptor antagonists	Structural/functional AI antagonists	Inhibition of receptor activation	Chen et al., 2011
	Signal trapping	Clathrate compound	AI sequestration	Taylor et al., 2009
	Suppression of LuxI/LuxR production	Interference RNA	Interference with the translation of LuxI/LuxR mRNA by non-coding small RNA	Chambers and Sauer, 2013

Numerous *in vitro* and *in vivo* experimental data (Brackman et al., 2011) on biofilm formation and antibiofilm unconventional strategies were reported (Roy et al., 2018), but their efficiency and safety need to be validated in clinical studies. The local delivery of QSIs in biofilms seems to be a promising lead, allowing a quick assessment of therapeutic efficiency. Therefore, developing appropriate local delivery systems and ways would be of most importance in future research. Despite the huge amount of data, only a few of the available QSIs are reaching the stage of clinical studies and, eventually, the bedside, and sometimes they have been approved for other biological activities, such as antimicrobial (e.g., azithromycin, which inhibits the alginate synthesis; vegetal extracts; natural compounds, which can also act as QSIs in subinhibitory concentrations) or antitumoral agents (Saurav et al., 2016; Rémy et al., 2018). There are also few patents using lactonase or acylase QSIs, proposed mainly as antibiofouling agents (Lee et al., 2013).

From the most challenging resistant species, known as ESKAPE (*Enterococcus faecium*, *Staphylococcus aureus*, *Klebsiella pneumoniae*, *Acinetobacter baumannii*, *Pseudomonas aeruginosa*, and *Enterobacter* species), and then changed to ESCAPE (*E. faecium*, *S. aureus*, *Clostridium difficile*, *A. baumannii*, *P. aeruginosa*, and Enterobacteriaceae), the gram-negative bacilli are the most problematic because of the lack of novel classes of antimicrobial agents efficient against these multiple- (MDR), extended (XDR), and pan-drug resistant (PDR) strains. From February 2017, these emerging MDR bacteria are also listed as critical in the WHO (World Health Organization) priority pathogen list for the research and development of new antimicrobials^1^.

The most known investigated biofilm regulators and their described mechanisms for *Klebsiella* sp., *Acinetobacter* sp., and *Enterobacter* sp., which are less investigated MDR ESKAPE agents, are presented in Table 2.

**TABLE 2:** Mechanisms of known biofilm signals and regulators in *A. baumannii, Klebsiella* sp., and *Enterobacter* sp.

Microorganism	Biofilm signals and regulators	Mechanisms	References
*Acinetobacter* sp.	– Csu assembly system composed from pilin subunits CsuA/B, CsuA, CsuB, and CsuE and transport proteins CsuC and CsuD – OmpA (Outer membrane protein A) – Biofilm-associated protein (Bap) – β-lactamase blaPER-1 – *pil* operon, codifying for type IV pili, *pap* operon, and *prpABCD* operon codifying also for pili – Poly-β-(1,6)-*N*-acetylglucosamine (PNAG)	– biofilm formation, adherence to the inert surfaces in many biofilm forming *Acinetobacter baumannii* strains, – biofilm formation, adherence of the strains to inert surfaces and host cells, – adherence to bronchial cells and for biofilm structure integrity, – increases adherence to the substratum, – involved in adherence and biofilm formation, – extracellular polysaccharide; function as intercellular adhesin within the biofilm.	Brossard and Campagnari, 2012; Yang et al., 2019; Colquhoun and Rather, 2020
	– AbaI/R QS system – BfmRS two component system – AdeRS, GacSA two component systems – cyclic di-GMP	– regulate biofilm formation, – biofilm master regulator, involved in regulation of *csu* operon and genes important for virulence and desiccation tolerance, – regulates pili synthesis, motility, biofilm formation, – regulate signaling in biofilms.	Ahmad et al., 2020; Colquhoun and Rather, 2020
*Klebsiella* sp.	– type 3 fimbriae (subunit MrkA, a chaperone-usher system MrkBC, the fimbrial tip adhesin MrkD, and MrkF) – CPS (capsular polysaccharides) – second messenger cyclic-di-GMP (c-di-GMP) – MrkH and MrkI transcriptional activators (encoded by *mrkHIJ* gene clusters) – MrkI – histone-like nucleoid-structuring protein (H-NS), CRP – ferric uptake regulator (Fur) – RcsAB (a two-component regulator of capsule synthesis) – IscR (iron-sulfur cluster regulator) – AI-2 interspecies QS system	– mediate stable adherence in biofilm, – involved in cell-to-cell communication and biofilm architecture, – biofilm regulation by control of type 3 fimbrial production (decrease concentration of c-di-GMP decreased the expression of the *mrkABCDF* preventing the synthesis of the type 3 fimbriae), – control c-di-GMP dependent phenotypes, – act as functional c-di-GMP phosphodiesterase and conduct to hydrolysis of c-di-GMP repressing type 3 fimbriae expression and biofilm formation, – control of type 3 fimbriae expression, – type 3 fimbriae expression, capsula and biofilm formation in *K. pneumoniae*, – regulate transcription of *galF* gene (controlling the biosynthesis of capsular polysaccharide) by binding to the *galF* promoter DNA, – modulate the iron-acquisition system and attachment, – regulate biofilm formation and LPS synthesis in *K. pneumoniae* biofilm by increase in the expression of two LPS-synthesis – related genes, *wbbM* and *wzm*.	Johnson et al., 2011; [Bibr B144][Bibr B42]; Johnson et al., 2011; Lin et al., 2017; Peng et al., 2018; Zhang et al., 2021
*Enterobacter* sp.	– ‘curli fimbriae’ – *the second* type VI secretion system (T6SS-2) – mRNA expression level of *csgA* and *csgD* genes (curli biogenesis genes).	– protein extracellular fibers involved in host cell adhesion and invasion, control the formation and architecture of *E. cloacae* biofilms, modulate adherence to abiotic and biotic surfaces, – regulate biofilm formation in *Enterobacter* sp.	Mezzatesta et al., 2012; [Bibr B173][Bibr B171]

Biofilm-associated infections often involve ESKAPE pathogens as etiological agents. Therefore, the purpose of this paper is to reveal and discuss the progress on the development of antibiofilm and anti-QS strategies in the less investigated gram-negative ESKAPE pathogens, such as *A. baumannii*, *K. pneumoniae*, and *Enterobacter* sp., and their potential contribution to the personalized control of infections produced by these emerging opportunistic pathogens.

## QS Signaling and Microbial Biofilms in *Acinetobacter* sp., *Klebsiella* sp., and *Enterobacter* sp.

Among the most dangerous threats concerning infection control gathered under the acronym ESKAPE, some of them are less investigated than the well-known QS experimental models, such as *P. aeruginosa* and *S. aureus*. However, the increasing incidence in the etiology of hospital-acquired infections and BAIs as well as the multiple intrinsic and acquired resistance mechanisms of the gram-negative species from the ESKAPE group, *Acinetobacter* sp., *Klebsiella* sp., and *Enterobacter* sp., justify the urgent need for the development of novel and effective antimicrobial strategies to target them. Table 3 summarizes some of the recent approaches investigated for BAI management in *A. baumannii*, *Klebsiella* sp., and *Enterobacter* sp.

**TABLE 3:** Recent approaches for BAIs management in *A. baumannii, Klebsiella* sp., and *Enterobacter* sp.

Approach	Microorganism	Mechanism/Effect	References
Bacteriophages	*Klebsiella* sp.	The ZCKP1 phage reduces biofilm biomass via soluble exopolysaccharide depolymerase, that has the ability to disrupt the capsule of *Klebsiella*, rendering it more susceptible to antibacterial agents	Taha et al., 2018
		Siphoviridae phage Z reduces the biofilm biomass after 24 and 48 h	Jamal et al., 2015
		Phage vB_KpnS_Kp13 drastically reduces the biofilm biomass (by ∼73%) after 48 h	Horváth et al., 2020
	*Acinetobacter baumannii*	The vB_AbaM_ISTD phage (Myoviridae family) reduces planktonic and biofilm-associated viable bacteria in a time-dependent manner	Vukotic et al., 2020
		The bacteriophage vB_AbaM-IME-AB2 infected and disrupted thebiofilm	Liu Y. et al., 2016
	*E. cloacae/E. asburiae*	The highly virulent bacteriophage N5822, isolated from an environmental source, reduced a preformed static host biofilm, and inhibited the formation of new biofilm by up to 90%	Nair et al., 2019
Low-frequency ultrasound (LFU)	*K. pneumoniae*	The treatment has increased the antimicrobial effect of with antimicrobial agents (meropenem, tigecycline, fosfomycin) in biofilm M-LFU (multiple –LFU) increased the duration of the synergistic effect as compared with S-LFU (single –LFU)	Liu et al., 2020
LFU	*A. baumannii*	LFU in combination with colistin and vancomycin may be useful in treating pan-resistant infections	Liu X. et al., 2016
Photodynamic inactivation (PDI) combined with chitosan	*A. baumannii*	A notable decrease of the number of viable biofilm cells	Fekrirad et al., 2021
Cathodic voltage controlled electrical stimulation (CVCES)	*A. baumannii*	The treatment has significantly reduced the implant-associated colony forming units (CFU) by over 91% and bone-associated CFU by over 88%	Ehrensberger et al., 2016
DNase I Dispersin B	*Klebsiella pneumoniae*, *Acinetobacter baumannii*	Biofilm-disrupting activity	Fleming and Rumbaugh, 2017
Synthetic, modified antimicrobial peptide 1018	*A. baumannii*, *K. pneumoniae, Enterobacter* sp.	Degradation of the (p)ppGpp bacterial stringent response signal	de la Fuente-Núñez et al., 2015; Wang et al., 2015; Wolfmeier et al., 2018
DJK-5, DJK-6 synthetic, D-enantiomeric, protease-resistant peptides			
Formulation of imipenem and silver NP	*A. baumannii*	Eradicated biofilms	Hendiani et al., 2015
Nanostructured Graphene- and Hexagonal Boron Nitride-Coated Surfaces	*Enterobacter cloacae*	Reduced biofilm formation	Zurob et al., 2019

*Acinetobacter* spp. is one of the hospital “superbugs,” considered today the most important nosocomial pathogen and the first priority on the WHO pathogen list requiring novel antibiotics, mainly due to its tolerance to desiccation, MDR mechanisms, and ability to develop medical device BAIs. Biofilm-forming ability seems to be much higher in clinical than in environmental isolates. The ability of *A. baumannii* clinical strains to form biofilms on abiotic substrata and epithelial cells increases their genetic resistance. Thus, at least 92% of the biofilm-forming nosocomial isolates seem to be MDR (Babapour et al., 2016), while an increased detection rate and expression of the blaPER-1 gene encoding for beta-lactam resistance were recorded in biofilm-forming isolates (Lee et al., 2008; Gaddy and Actis, 2009). It was reported that CarO and OmpA outer membrane proteins are interacting physically with the OXA-23 carbapenemase, leading to an enhanced carbapenem resistance by cumulating non-enzymatic and enzymatic resistance mechanisms (Chopra et al., 2013).

Recent genomic studies highlight the presence of much greater virulence determinants in *A. baumannii* than previously thought. The virulence genes, including those involved in biofilm formation and the current progress in developing antibiofilm agents in *A. baumannii*-derived infections, are summarized by Eze et al. (2018). The success of *A. baumannii* in host colonization mainly depends on its adherence capacity, but other virulence factors are also incriminated. These include: K1 capsular polysaccharides, surface antigen protein 1, outer membrane porins (which are involved in adhesion, biofilm formation, and drug resistance), Bap (biofilm-associated protein), inflammatory cytokine induction molecules (Eze et al., 2018; Harding et al., 2018), iron transport systems and siderophores (such as acinetobactin), poly-(1-6)-*N*-acetylglucosamine (PNAG, which is one of the most important structures for biofilm formation correlated with higher resistance), activation of phosphomannomutase/phosphoglucomutase (*algC*) gene [encoding for alginate and lipopolysaccharide (LPS) during biofilm development and correlated with the MDR level], type I chaperone-usher pilus system (Csu pili) regulated by QS (which is critical for the adherence to inert substrata), LHp2_11085 factor involved in adherence to inert and cellular substrata, and A1S_0114 regulatory gene of surface proteins and pili-assembly system expression (Shin et al., 2009; Xiang et al., 2012; Zarrilli, 2016; Álvarez-Fraga et al., 2017). The enormous adaptability of resistant strains, supported by the acquisition and dissemination of resistance and virulence markers, renders it a dangerous opportunistic pathogen, particularly in the case of immunosuppressed patients from the intensive care units (Vrancianu et al., 2020b,c).

**FIGURE 1:**
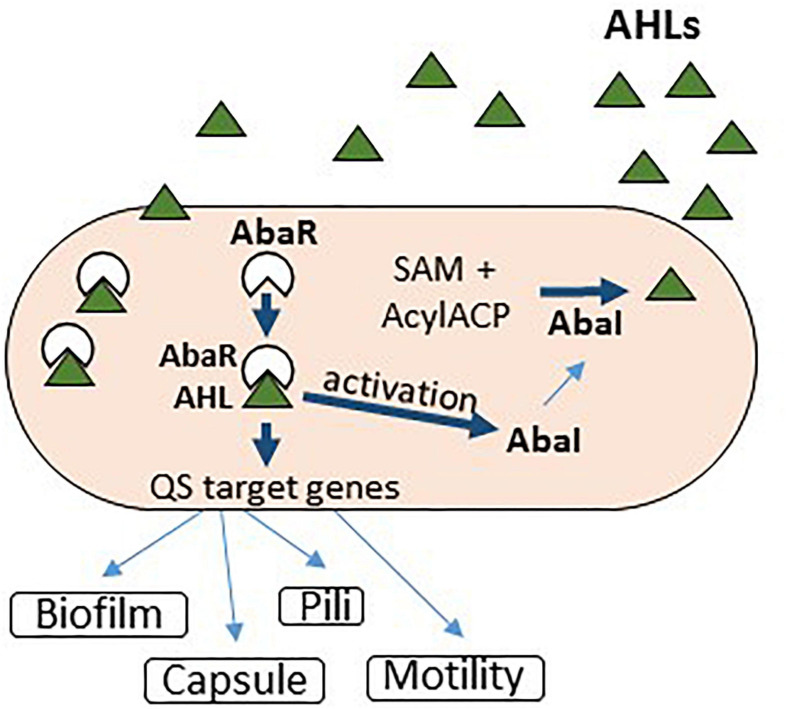
Quorum sensing signaling in *Acinetobacter baumannii.*

The QS system in *Acinetobacter* sp. has been described as homologous to the LuxR receptor (AbaR) and LuxI synthase (AbaI) system from *V. fischeri*. However, phylogenetic studies indicate that its QS genes (*abaI* and *abaR*) were acquired horizontally from *Halothiobacillus neapolitanus* (Bhargava et al., 2010, 2015a). More than 63% of the *Acinetobacter* spp. analyzed strains produced more than one AHL (≥C10), including *N*-(3-hydroxydodecanoyl)-L-homoserine lactone (OH-dDHL). The QS mechanism plays an important role in *Acinetobacter* spp. motility, expression of multidrug efflux pumps, and biofilm development (Figure 1). However, little is known about the cascade of genes associated with various mechanisms controlled by the QS system in *A. baumannii* (López et al., 2017). Iron limitation seems to regulate the expression of virulence and QS factors in *A. baumannii* clinical strains, including the biofilm development capacity (Kim H. W. et al., 2013; Modarresi et al., 2015). In their turn, the QS signaling molecules could chelate iron, inducing the occurrence of the stress response (Minandri et al., 2016). This could explain the persistence of *A. baumannii* biofilms in iron-depleted environments. Siderophores can chelate iron, zinc, copper, and other metals, interfering thus with the activity of antibiotics and host molecules while modulating the oxidative stress (Figure 1).

Studies related to the inhibition of QS in *Acinetobacter* are limited. The lack of QSIs for this clinically significant pathogen is a mounting concern, especially with the increasing frequency of MDR strains (Subhadra et al., 2016).

**FIGURE 2:**
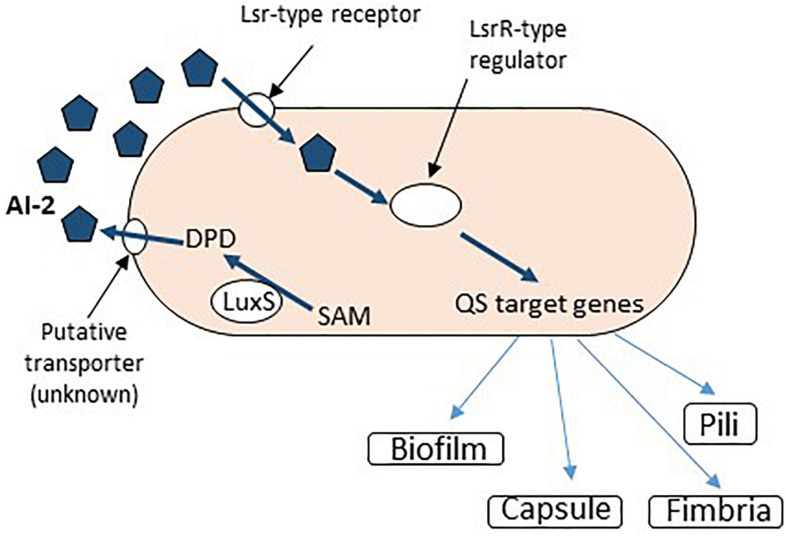
AI-2 dependent QS signaling in *Klebsiella* sp. SAM, *S*-adenosyl methionine; DPD, 4,5-dihydroxy-2,3-pentanedione.

*Klebsiella pneumoniae* is a versatile opportunistic pathogen, exhibiting many virulence features, allowing it to colonize different inert substrata, including the urinary catheters, such as fimbriae (of type 1 and 3), capsular polysaccharides, factors involved in aggregative adhesion, and siderophores (Stahlhut et al., 2012; Vuotto et al., 2014; Paczosa and Mecsas, 2016). If in the preantibiotic era *K. pneumoniae* was considered an important etiological agent of community-acquired (CA) infections (such as severe pneumonia in debilitated patients), presently, because of its high resistance to last-resort antibiotics, such as carbapenems and colistin, the spectrum of *K. pneumoniae* infections has broaden, including CA and healthcare-associated life-threatening infections (Lederman and Crum, 2005). *K. pneumoniae* is involved in 5–7% of all healthcare-associated infections (Clegg and Murphy, 2016). *K. pneumoniae* uses QS TCSs to control the host–pathogen interactions and to coordinate the virulence and the AR mechanisms, including the rapid development of biofilms on abiotic surfaces (Balestrino et al., 2005; Srinivasan et al., 2012; Tiwari et al., 2017). The QS system in *K. pneumoniae* is mediated by the AI-2 AI encoded by a homolog of *luxS* from *V. harveyi* (Figure 2), and also by *N*-octanoyl homoserine lactone (C8-HSL) and *N*-3-dodecanoyl-L-homoserine lactone (C12-HSL) (Balestrino et al., 2005; Yin et al., 2012). Mutations in *luxS* are correlated with an increased expression of two LPS synthesis-related genes, *wbbM* and *wzm*, also involved in biofilm formation (Sun et al., 2016). Recent data confirm the involvement of the AI-2 QS system in the expression of LPS and PNAG biosynthesis, as well as biofilm development of an extensively drug-resistant *K. pneumoniae* clinical isolate (Chen et al., 2020; Figure 2).

**FIGURE 3:**
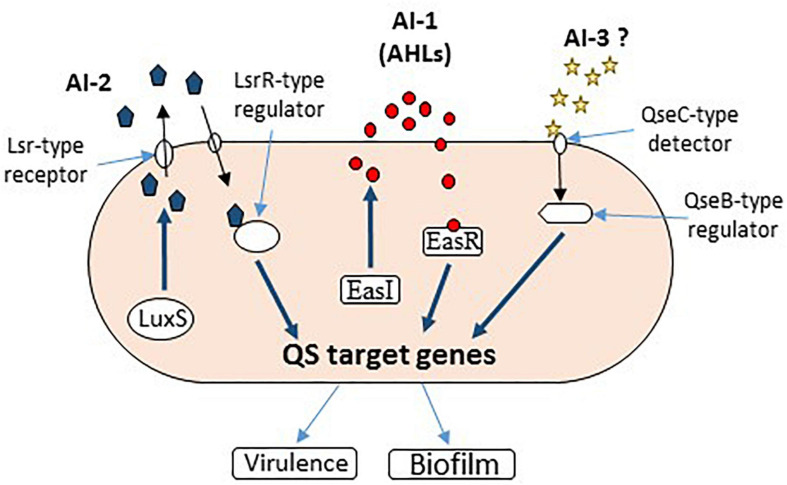
Quorum sensing signaling in *Enterobacter* sp. All three AIs have been reported to function in *Enterobacter* sp. (AI-1, AI-2, and AI-3).

*Enterobacter* genus, especially through its two most prominent species, *Enterobacter aerogenes* and *Enterobacter cloacae*, is a versatile nosocomial pathogen with serious implications in respiratory and urinary tract infections (Sanders and Sanders, 1997). Unfortunately, little is known about quorum control and pathogenesis in this group of bacteria. Most of the available data come from food-associated studies. It uses C4 and C6-HSLs as QS signaling molecules (Yin et al., 2012; Lau et al., 2013), encoded by a LuxR homolog, which has been found to negatively regulate bacterial adhesion and biofilm formation (Shankar et al., 2013). AI-2-mediated QS has also been suggested to play a role in intercellular communication within *Enterobacter* spp. (Figure 3), as Lsr-type receptors have been found in strains of *Enterobacter cancerogenus*, *E. cloacae*, and *Enterobacter mori* (Rezzonico et al., 2012*;* Tay and Yew, 2013). The AI-3 activity, initially reported in enterohemorrhagic *E. coli O157:H7*, was also detected in *E. cloacae* isolated from normal microbiota (Reading and Sperandio, 2006).

A recent study documented the cloning and characterization of a transcriptional regulator, luxR homolog from *Enterobacter asburiae*, as well as the functionality and specificity of EasR protein in response to different AHL signaling molecules to activate gene transcription from QS target promoters (Figure 3). However, further genome-wide comparative transcriptomics are needed to elucidate the possible roles of QS, especially in the pathogenicity of different *Enterobacter* spp. (Lau et al., 2020; Figure 3).

## Biofilm and QS Modulators in *Acinetobacter*, *Klebsiella*, and *Enterobacter*

As BAIs produced by ESKAPE pathogens are currently very difficult to treat, the modulation of key molecular mechanisms of biofilm development, including the QS signaling, represents a very promising alternative in handling such infections (Lazar and Chifiriuc, 2011).

Below, we present the results of different studies that have reported the use of natural and synthetic compounds or other mechanical, physical, or biological strategies to modulate QS and inhibit biofilm development in *Acinetobacter*, *Klebsiella*, and *Enterobacter* species.

### Antibiotics and Antiseptics

When used in specific amounts, antibiotics could act as intermicrobial signaling agents, impacting on the biofilm homeostasis, motility, and type three secretion system (TTSS) (Linares et al., 2006). Subinhibitory concentrations of antibiotics modulate the QS mechanism, in contrast to bactericidal effects observed at high concentrations (Rémy et al., 2018). Some antibiotics (i.e., streptomycin, gentamicin, and myomycin), utilized in subinhibitory concentrations, have been found to inhibit QS signaling in *A. baumannii*. Streptomycin can act as an antagonist of AbaR and inhibits QS in *A. baumannii* by downregulating the *abaI* gene, encoding for the AI synthase, resulting in the corresponding decrease in 3-oxo-C12-HSL production (Saroj and Rather, 2013). It has been shown that subinhibitory concentrations of trimethoprim-sulfamethoxazole completely inhibit the pilin expression in *A. baumannii*, disrupting the biofilms formed on inert substrata and promoting a planktonic lifestyle, with bacterial cells more susceptible to antimicrobial agents (Harding et al., 2018).

In catheter-associated *A. baumannii* infections, the combination of colistin–levofloxacin [at 400 × minimum inhibitory concentration (MIC) each] or a combination of colistin/tigecycline/levofloxacin at 400 × MIC with clarithromycin (200 mg/ml) and/or with heparin (1,000 U/ml) as lock solutions proved to be therapeutically effective against BAIs (Ozbek and Mataraci, 2013). Tigecycline, imipenem–rifampicin, and colistin–rifampicin (Song et al., 2015), as well as sulbactam–tigecycline and meropenem–sulbactam, proved to be effective against *A. baumannii* biofilms, decreasing the biofilm mass and thickness (Wang et al., 2016).

In associated wound infections, drug-resistant *A. baumannii* forms biofilms, which are very recalcitrant to topical antibacterial agents. However, when associated with ambroxol, a respiratory mucus secretolytic agent, the topical antibacterial agents proved to be effective against wound-associated BAIs (Huang et al., 2012).

A number of Food and Drug Administration (FDA)-approved drugs with different pharmacological activities, including erythromycin (antibiotic), chloroquine (antimalarial), levamisole (antiparasitic), and propranolol (adrenergic blocker), were recently proven to interfere with QS and virulence in MDR *A. baumannii* clinical isolates. These drugs repressed the expression of *abaI* gene *in vitro* and showed significant virulence repression in *A. baumannii*, both *in vitro* and *in vivo*, expressed by improved mice survival rates. In addition, molecular docking studies against AbaI and AbaR proteins of QS system in *A. baumannii* revealed the potential inhibition of QS by these drugs (Seleem et al., 2020).

Oxidizing biocides, such as sodium hypochlorite and hydrogen peroxide (Ceni et al., 2020) proved to be more efficient against biofilms than non-oxidizing ones (e.g., sulfathiazole, glutaraldehyde) (Shakeri et al., 2007), with single species *Acinetobacter* biofilms proving to be more susceptible than polymicrobial ones (Runci et al., 2016).

### Natural QS Modulators

Quorum sensing inhibition was first observed in natural habitats (Pietschke et al., 2017). The QSIs were synthesized either by other organisms to protect from pathogenic bacteria or by bacteria to gain a survival advantage (Saurav et al., 2017). The concept of QS modulators includes numerous types of natural or synthetic molecules, but also phages and cells, like quorum quenching (QQ) bacteria.

The natural QSIs are used as a backbone to obtain synthetic QSIs. As antipathogenic drugs, the QSIs could be used either to treat or prevent infections and synergize with current antibiotics and anti-infectious immune effectors (Romero et al., 2012; Tang and Zhang, 2014; Pietschke et al., 2017).

QSIs agents are diverse, such as exogenous AI-2, the AIP type I, RNAIII-inhibiting peptide, benzamide–benzimidazole “M64” derivative (Starkey et al., 2014), or plant/microbial-derived compounds, i.e., essential oils (Saviuc et al., 2015), usnic and barbatic acids—lichen secondary metabolites (Francolini et al., 2004; Chifiriuc et al., 2007; Grumezescu et al., 2015), 6-gingerol (Kim H. A. et al., 2015; Kim H. S. et al., 2015), solenopsin A, catechin, ellagic acid derivatives, curcumin, diterpenoide lactone 14-alpha-lipoyl and rographolide (Zeng et al., 2011), ajoene (Jakobsen et al., 2012), patulin and penicillic acid isolated from *Penicillium* sp. (Rasmussen et al., 2005a,b), probiotic culture supernatants and purified compounds (Ditu et al., 2011; Cotar et al., 2013), and enzymes (*Bacillus* spp.-derived lactonase) (Kiran et al., 2011). Natural QSIs represent an ecological and intelligent way to fight microbial pathogens efficiently without exhibiting the side effects normally associated with antibiotics.

Microbial metabolites produced by drinking water bacteria proved to inhibit *Acinetobacter calcoaceticus* biofilms (Simões et al., 2008). The 5-episinuleptolide, a natural compound isolated from *Sinularia leptoclados* inhibited the *A. baumannii* biofilm development as well as the MDR *A. baumannii* strains by decreasing the poly-PNAG expression (Tseng et al., 2016). There are reports on utilizing several natural compounds as QSIs that interfere with AHL receptors in *A. baumannii*, including patulin, clavacin, vanillin, and alliin (Cady et al., 2012). Linalool, a major compound *Coriandrum sativum* essential oil, flavonoids from *Glycyrrhiza glabra*, and *Salvia glutinosa* essential oil have been shown to exhibit QSI as well as anti-*A. baumannii* virulence and biofilm activity (Bhargava et al., 2015b; Alves et al., 2016; Tutar, 2016; Shaaban et al., 2019). Vegetal QSIs have also been proposed to be used as natural food preservatives to prevent opportunistic food-borne infections. Petunidin, a dark-red or purple water-soluble pigment found in many red berries (an *O*-methylated anthocyanidin of the 3-hydroxy type) at sub-MIC values, drastically reduced the EPS production in *K. pneumoniae*, the antibiofilm effect being much enhanced when acting synergistically with conventional antibiotics. Molecular modeling studies predicted that petunidin induces changes in the 3D structure of the LasR receptor protein, suggesting that it acts as an effective competitive inhibitor of QS signaling through the LasR receptor pathway (Gopu et al., 2015). The essential oil from cumin seeds reduced biofilm formation in *K. pneumoniae*, but without any direct connection with QS pathway inhibition.

Lactonases are natural enzymes able to degrade AHL-type AIs. Two clusters of AHL lactonases were described in prokaryotes: AiiA and AttA. The AiiA lactonase has been shown to decrease the number of *E. cloacae* cells during early biofilm formation in continuous biofilm models, while flagellin and outer membrane protein expressions were downregulated (dos Reis Ponce et al., 2012; Kim I. H. et al., 2013). AttA cluster was described in *K. pneumoniae* and regulates the fermentative metabolism and virulence in this bacterium (Sun et al., 2016).

Engineered and natural lactonases (e.g., a thermostable engineered mutant of phosphotriesterase-like lactonase from *Geobacillus kaustophilus*, with enhanced catalytic activity on different AHLs ranging from 6 to 12 carbons) induced a significant decrease in *A. baumannii*-associated biofilm development (Chow et al., 2010, 2013, 2014). The large AHL spectrum of these enzymes proves their promising potential to fight infections associated with various gram-negative bacteria using AHL-mediated QS signaling. It has also been reported by Zhang et al. (2017) that the recombinant enzyme, MomL, is also able to degrade QS molecules, and thus reducing the biofilm formation and increasing the *in vitro* susceptibility of biofilm cells to different antibiotics of some *Acinetobacter* sp. strains and of *P. aeruginosa* PAO1. However, the results were strain dependent, and when this enzyme was tested against polymicrobial biofilms and wound-associated biofilm infections, it was ineffective, probably due to the fact that the *in vivo* conditions could affect the stability of the enzyme and its penetration through the biofilm matrix (Zhang et al., 2017).

Antimicrobial peptides (AMPs) are considered promising candidates for developing antimicrobial and anti-inflammatory agents and an example of how the natural antimicrobial strategies from the living world could be exploited or mimicked to create effective antimicrobial drugs (Aminov, 2010). It was demonstrated that the AMP LL-37 disrupted the structure of *A. baumannii* biofilms at low concentrations of 2.5 μg/ml (Shin et al., 2009). Also, a natural AMP complex (defensin, cecropin, diptericin, and proline-rich peptide families that are produced during bacterial infections) from the blow fly maggot *Calliphora vicina* has been proven to be active both on the cellular and matrix components of *A. baumannii* biofilms, at the same time lacking toxicity toward human immune cells (Gordya et al., 2017). Magainin 2 AMP has been proven to be an effective treatment for *A. baumannii* infections (Kim et al., 2018). Many other AMPs, such as CAMEL (a hybrid AMP consisting of cecropin from *Hyalophora cecropia* and melittin from *Apis mellifera*), pexiganan, cecropins identified in *Musca domestica*, and myxinidin isolated from *Myxine glutinosa*, revealed antibiofilm activity against resistant *A. baumannii* strains (Han et al., 2017). Natural AMPs can be a starting point for the biosynthesis of AMPs with similar functions, being an attractive therapeutic option for preventing and controlling *A. baumannii* BAIs (Vrancianu et al., 2020a).

### Synthetic Modulators

Antagonists of diguanylate cyclase enzyme that synthesize c-di-GMP, a second messenger signal essential for biofilm formation, were proven to inhibit QS and biofilm formation in *A. baumannii* (Sambanthamoorthy et al., 2014). Recently Subhadra et al. (2016) reported the efficacy of virstatin, a small organic molecule, as an inhibitor of biofilm formation and motility in *A. baumannii* and *Acinetobacter nosocomialis*, acting by inhibiting the anoR/I signaling pathway (Subhadra et al., 2016). Various non-native AbaR ligands inhibited AHL-mediated QS and, subsequently, *A. baumannii* surface motility and biofilm formation (Stacy et al., 2012). One of the strongest AbaR antagonists (with very low IC50 values less than 20 μM) largely contained aromatic acyl groups, whereas the AbaR agonists closely resembled OH-dDHL (Stacy et al., 2012). A 2-aminoimidazole-based antibiofilm agent proved to effectively decrease biofilm development on indwelling medical devices (Peng et al., 2011). The dihydrofolate reductase inhibitor *N2*, *N4*-disubstituted quinazoline-2,4-diamines, has been shown to decrease by 90% the number of biofilm-embedded cells at concentrations similar to MIC, being more effective than tigecycline (Fleeman et al., 2017).

Synthetic AI-2 interferes with QS modulated phenotypes in *K. pneumoniae*, restoring acetoin, ethanol, and acetic acid production in *luxS* knockout mutant (Sun et al., 2016).

### QSI–Antibiotic Synergic Combinations

QSIs often exhibit a synergic antibiofilm activity with antibiotics (Algburi et al., 2017). Scientists suggest using furanone in combination with antibiotics; this approach being more acceptable by patients (Grandclément et al., 2015). The antivirulence compounds affecting the cell wall composition may render bacteria more susceptible to antibiotics; therefore, the association of antivirulence agents with current antibiotics could be anticipated as efficient against biofilms (Escaich, 2010). The QS-controlled bacterial adherence and colonization could be inhibited using novel inhibitors of pili synthesis, represented by sortases or specific inhibitors of TTSS. Synergic combinations of antibiotics and QSIs are expected to be soon evaluated for QS modulation in *A. baumannii, Klebsiella* sp., and *Enterobacter* sp.

### Nanomaterials

Nanotechnology offers promising leads for fighting BAIs by developing nanoantimicrobials and antibiofilm materials and by improving the drug loading and the controlled release of antimicrobial agents into biofilms. Numerous nanostructured materials have been developed to target biofilm pathogens, including the less investigated ESKAPE gram-negative species discussed in this study. Liposomes of different compositions proved to be efficient carriers for ciprofloxacin and meropenem against *K. pneumoniae* biofilms (Gubernator et al., 2007) and polymyxin B/clarithromycin against *A. baumannii* and *Acinetobacter lwoffii* biofilms (Khan et al., 2016; Halbus et al., 2017).

Gallium nitrate is a potent inhibitor of *A. baumannii* biofilm formation and a disruptor of mature biofilms developed in human serum, probably also due to iron depletion in the multicellular communities formed by *A. baumannii* (Runci et al., 2016).

Metallic nanoparticles (NPs) have a great potential for antimicrobial applications, exhibiting multiple mechanisms of action, such as membrane lesions induced by direct contact or indirectly, by the release of free metal ions, protein inactivation, nucleic acid damages, and release of reactive oxygen species (ROS) (Grumezescu et al., 2015; Samrot et al., 2020; Tripathy et al., 2020). They could also exhibit synergic action with the host immune effectors. Metallic NPs can also be associated with the current antibiotics or other pharmaceutically active compounds to overcome the resistance threat, particularly in hospital settings (Tudose et al., 2015a,b, 2016). The small size and tailored properties of NPs seem to represent an advantage for penetrating more efficiently the biofilm matrix (Holban et al., 2016; Abdelghany et al., 2020). Silver NPs alone and associated with biocides or antibiotics (imipenem) proved very active on *A. baumannii*, both planktonic and biofilm growth (Hendiani et al., 2015). Zinc oxide NPs were also reported to impact the biofilm formation of different gram-positive and gram-negative pathogens, being considered future nanoantibiotics (Visinescu et al., 2015, 2018; Muzammil et al., 2018). It has also been shown that *K. pneumoniae* uropathogenic strains isolated from complicated urinary tract infections have shown decreased adherence to silver-treated silicone or latex catheters (Gabriel et al., 1995). Titanium NPs also show promising antibacterial and antiadhesive properties against *A. baumannii* and *K. pneumoniae* strains (Ibrahem et al., 2014).

The medical device-associated infections could be prevented by developing antiadherent materials or coatings. For example, “biospecific polymers” coated with antiadhesive molecules or doped with inhibitory biofilm-associated gene expression could represent an alternative (Pascual, 2002). Urinary catheters coated with nitrofurazone proved to have an enhanced resistance to biofilm development by ESKAPE urinary pathogens (Johnson et al., 2012; Zhu et al., 2019).

### Biofilm Dispersal Diffusible Signal Factors

Bacteria can induce dispersal to escape from the biofilm macrostructure in response to a broad range of input signals. The biofilm dispersal process is the starting point of systemic infections since it triggers the release of bacteria into the host (Marks et al., 2013; Guilhen et al., 2017). Therefore, knowledge of the regulation of dispersal factors would help control the development of BAIs and the systemic spread of bacteria. It is known that biofilm dispersal could be triggered by (i) environmental factors [i.e., availability of iron (Musk et al., 2005; Glick et al., 2010), carbon source (Uppuluri et al., 2010; Bonnichsen et al., 2015), presence of heavy metals (Petrova et al., 2015), temperature (Nguyen et al., 2015), pH (Uppuluri et al., 2010), and oxygen limitation (An et al., 2010)] and (ii) bacteria and host-produced signaling molecules [i.e., AHLs, AIPs, diffusible signal factors (DSFs) (Ueda and Wood, 2009; Tao et al., 2010; Periasamy et al., 2012), human intestine epithelial cell signals (Sanchez et al., 2016), and nitric oxide (Li et al., 2013)].

Diffusible signal factors was originally found in *Xanthomonas campestris* and is a new family of widely conserved QS fatty acid signals in gram-negative bacteria, which regulate biofilm formation, motility, virulence, and AR (Deng et al., 2014). Studies showed that DSFs act as interspecies biofilm regulators since, for example, DSF produced by *P. aeruginosa* can disperse biofilms of other gram-negative (i.e., *E. coli*, *K. pneumoniae*, and *Proteus mirabilis*), gram-positive (i.e., *Streptococcus pyogenes*, *Bacillus subtilis*, *S. aureus*), but also yeast (*Candida albicans*) strains (Davies and Marques, 2009). However, little is known regarding DSF regulation in the development and dispersal of *K. pneumoniae*, *A. baumannii*, and *Enterobacter* sp. biofilms. Recent studies showed that DSFs and other fatty acids inhibit key virulence mechanisms, such as planktonic growth, capsule production, and cell adhesion and induce biofilm dispersal in *K. pneumoniae* (Rahmani-Badi et al., 2014; Gupta et al., 2020; Kumar et al., 2020). Chowdhury et al. (2021) reported a *cis*-2-hexadecenoic acid (c2-HAD) DSF homolog encoding gene (*rpfF*) in *Enterobacter* sp. This DSF was proven to control the intestinal invasion of *Salmonella* sp. and control the main virulence regulon in this microorganism (Chowdhury et al., 2021). c2-HAD is supposed to interfere also with the virulence of other intestinal gram-negative bacteria, including *Enterobacter* species. Several mono-unsaturated chain fatty acids that could act as DSFs were demonstrated to affect QS communication and inhibit motility and biofilm formation of *A. baumannii* clinical isolates. These fatty acids decreased the expression of the regulator *abaR* from the LuxIR-type QS system. Consequently, they reduced the AHL production with a direct impact on biofilm dispersal (Nicol et al., 2018).

All the strategies proposed above are based on the molecular regulation of key phenotypes of virulence and resistance. These approaches are preferred in recent years since researchers believe that signaling modulation could prevent the development of the infectious process and the selection of resistant mutants compared to classical antibiotics. QS and biofilm control by such molecules could not interfere with population fitness but with some social behavior of microorganisms, which are key for their pathogenesis.

### CRISPR (Clustered Regularly Interspaced Short Palindromic Repeat) System

One of the most attractive leads to the fight against bacterial resistance is the CRISPR-Cas system, first described by Ishino et al. (1987). CRISPR-Cas is considered a bacterial immune defense system that could specifically recognize and degrade foreign nucleic acids. The CRISPR platform has been used to achieve rapid genomic editing by deletions, insertions, and point mutations, to investigate the oxidative stress (OxyR) mechanisms, as well as the *abaI* gene role in biofilm formation of *A. baumannii* (Wang et al., 2019; Vrancianu et al., 2020a).

**FIGURE 4:**
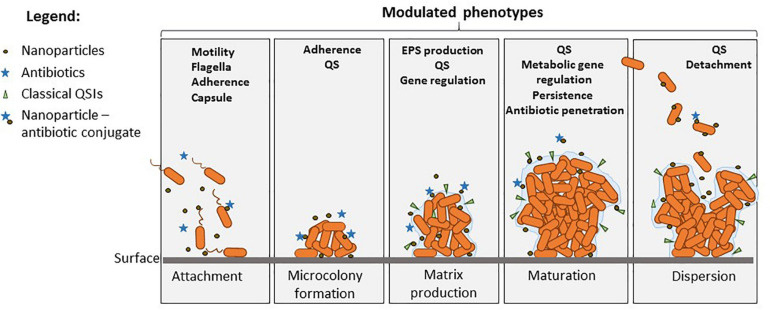
Overview of the modulated phenotypes in biofilms produced by *Acinetobacter*, *Klebsiella*, and *Enterobacter*, which could be modulated by diverse QS modulators, NPs, and antibiotics. Nanoparticles can inhibit the attachment and early biofilm development, being also able to deliver antibiotics, QSIs, and other bioactive molecules in the biofilm. Phenotypes such as motility and attachment are modulated by such molecules, and this controls biofilm initiation. Maturation is influenced by EPS inhibition and QS interference, which also impacts the metabolic changes in the biofilm such as stress response, tolerance, and persistence. Finally, dispersion of mature biofilms could be also controlled by some molecular inhibitors controlling cell detachment.

Studies suggested that certain bacteria employ the Cas proteins of CRISPR-Cas3 systems to target their own genes, which also alters the virulence during host invasion. It seems that numerous gram-negative bacteria use QS signaling to control adaptive immunity through the regulation of multiple CRISPR-Cas systems. Such interference has been revealed in *Serratia* sp., *Pseudomonas* sp. (Høyland-Kroghsbo et al., 2017; Noirot-Gros et al., 2019), and *Salmonella* sp. (Cui et al., 2020), where the tool is currently investigated to understand QS molecular signaling and biofilm formation. Cas3 deletion upregulated the *luxS* regulated operon related to QS and downregulated biofilm-forming-related genes in *Salmonella* interfering with pathogenicity island 1 genes related to the TTSS (Cui et al., 2020). Figure 4 shows the main phenotypes that impact on biofilm development and could be targeted for the management of ESKAPE infections.

## *In vivo* Models of Infection

Animal models are crucial to developing new therapeutics and vaccines and play critical roles in the assessment of understanding infection. Over the years, several animal models were developed for *A. baumannii*, *K. pneumoniae*, and *Enterobacter* sp. Pneumonia mouse models have been most widely developed in *A. baumannii*. However, most of the tested strains do not infect immunocompetent mice or induce only self-limiting pneumonia with no or very limited local and systemic dissemination. Other studies use immunocompromised (i.e., neutropenic) mice or treat mice with mucin or agar to increase host susceptibility to *A. baumannii* and bacterial virulence (van Faassen et al., 2007; Eveillard et al., 2010). Despite their limitation, these mouse models are useful for investigating bacteria virulence and the development of the infectious process. Recently, a mouse model of *A. baumannii*-associated pneumonia using a clinically isolated hypervirulent strain showed reliable reproduction of the most relevant features of human acute pulmonary infection and pathology (Harris et al., 2013). A similar model (Luna et al., 2019) was utilized to analyze the efficiency of antibiotic combinations in MDR *A. baumannii* pneumonia. The study demonstrated the synergistic effects of the combination of colistin with fosfomycin and minocycline, respectively, as therapeutic options in carbapenem-resistant *A. baumannii* mouse infection (Ku et al., 2019). Mouse models are also investigated to reveal *A. baumannii* strains and clones, which are associated with increased risk fatality and are circulating in the human population (Nutman et al., 2020).

*Caenorhabditis elegans* is another model developed for investigating *A. baumannii* virulence and biofilm development *in vivo*. The nematode is currently the preferred model for screening the infection, resistance, and virulence correlations in most clinically relevant *Acinetobacter* species (Cosgaya et al., 2019).

Few *in vivo* models were developed to study *K. pneumoniae* infection. Wax moth *Galleria mellonella* has been utilized to study key virulence mechanisms in *Klebsiella* sp., such as cell death associated with bacterial replication, avoidance of phagocytosis by phagocytes, the attenuation of host defense responses, and the production of antimicrobial factors. Numerous studies support the utility of *G. mellonella* as a surrogate host for assessing infections with *K. pneumoniae* (Insua et al., 2013). However, some research reports the better utility of murine models to investigate *K. pneumoniae* infection and host interaction. Along with their proven utility in the elucidation of pneumonia mechanisms, mouse models were recently used to evaluate *K. pneumoniae* gastrointestinal (GI) colonization and host-to-host transmission. Using an oral route of inoculation and fecal shedding as a marker for GI colonization, authors showed that *K. pneumoniae* can asymptomatically colonize the GI tract in immunocompetent mice and modifies the host GI microbiota. A hypervirulent *K. pneumoniae* isolate evaluated in that study was able to translocate from the GI tract and cause a hepatic infection that mimicked the route of human infection. Authors claim expression of the capsule is required for colonization. Also, treatment with antibiotics of infected mice led to changes in the host microbiota and the development of a transient supershedder phenotype, which enhanced transmission efficiency. Therefore, mouse model can be used to determine the contribution of host and bacterial factors toward *K. pneumoniae* dissemination (Young et al., 2020).

In *Enterobacter* sp., *in vivo* models of infection are scarce. *G. mellonella* larvae were recently studied to determine the antibacterial efficacy of various drugs and proved its utility also for the investigation of host–pathogen interactions in *E. cloacae*. The study concluded that *G. mellonella* killing significantly depends on the number of *E. cloacae* cells injected in a dose-dependent manner. Moreover, survival can be reduced by increasing the postinoculation temperature. Also, treatment of lethal *E. cloacae* infection with antibiotics with proven *in vitro* activity significantly prolonged the survival of larvae, as compared with antibiotics to which the bacteria were resistant. The therapeutic benefit arising from the administration of antibiotics was also correlated with a reduced burden of *E. cloacae* cells in the hemolymph (Yang et al., 2017).

Our study highlights that *G. mellonella* larvae proved to be the most investigated *in vivo* infection model for *A. baumannii* (Jeon et al., 2019), *K. pneumoniae*, and *Enterobacter* sp. (Cieślik et al., 2021). However, more models currently applied for other gram-negative bacteria (i.e., *Drosophila melanogaster*, zebrafish, mouse, rat) are expected to emerge in the near future in order to enhance knowledge regarding biofilm infections determined by less investigated emerging ESKAPE pathogens.

## Conclusion

To the best of our knowledge, this is the first paper focusing on the current progress in developing antibiofilm and anti-QS strategies for fighting the less investigated gram-negative ESKAPE pathogens: *K. pneumoniae*, *A. baumannii*, and *Enterobacter* sp.

The surveyed literature reveals some promising leads for the development of efficient strategies against these problematic superbugs, such as combinations of QSIs and/or antibiotics administered locally or with improved and controlled targeted delivery by using nanocarriers. Researchers are currently exploiting the great perspectives offered by CRISPR-Cas in the research of BAIs. It will probably soon be applied in the investigation of less analyzed ESKAPE pathogens.

A promising priority lead is represented by natural QSIs that could provide an ecological approach, with great therapeutical and preventive value, and can be used as the backbone to obtain synthetic, non-pollutant QSIs. This approach will foster the development of social microbiology, which will exploit the antagonistic biological relationships for finding attractive and intelligent anti-infectious strategies.

The development of QS modulation strategies for clinically significant biofilm-producing pathogens such as *K. pneumoniae*, *A. baumannii*, and *Enterobacter* sp. could be of mounting importance for effectively controlling the nosocomial and CA BAIs, especially with the continuing evolution of MDR, XDR, and PDR strains. QS modulators are also less likely to select for resistance and eventually would have fewer side effects and ecotoxicity.

*In vivo* models are very useful to decipher molecular mechanisms during infection and also the utility of newly developed agents aiming to control virulence, biofilm modulation, and resistance of less investigated ESKAPE bacteria. More and specific infection models are expected to emerge in the next few years.

## Author Contributions

All authors listed have made a substantial, direct and intellectual contribution to the work, and approved it for publication.

## Conflict of Interest

The authors declare that the research was conducted in the absence of any commercial or financial relationships that could be construed as a potential conflict of interest.

## Publisher’s Note

All claims expressed in this article are solely those of the authors and do not necessarily represent those of their affiliated organizations, or those of the publisher, the editors and the reviewers. Any product that may be evaluated in this article, or claim that may be made by its manufacturer, is not guaranteed or endorsed by the publisher.

## Footnotes

1
https://www.who.int/medicines/publications/WHO-PPL-Short_Summary_25Feb-ET_NM_WHO.pdf?ua=1
